# Association between motor symptom severity and urinary dysfunction in Parkinson’s disease: a retrospective study

**DOI:** 10.3389/fnagi.2025.1688656

**Published:** 2025-12-12

**Authors:** Jun Seok Lee, Joonsang Yoo, Nak-Hoon Son, Hye Jin Byun, Sooyeoun You

**Affiliations:** 1 Department of Neurology, Dongsan Hospital, Keimyung University School of Medicine, Daegu, Republic of Korea; 2 Department of Neurology, Yongin Severance Hospital, Yonsei University College of Medicine, Yongin, Republic of Korea; 3 Department of Statistics, Keimyung University, Daegu, Republic of Korea; 4 Department of Urology, Dongsan Hospital, Keimyung University School of Medicine, Daegu, Republic of Korea; 5 Department of Neurology, Seoul Medical Center, Seoul, Republic of Korea

**Keywords:** Parkinson’s disease, urinary dysfunction, overactive bladder, motor severity, quality of life

## Abstract

**Introduction:**

Urinary dysfunction is a common non-motor symptom in patients with Parkinson’s disease (PD) and is often associated with greater motor disability and reduced quality of life. Despite its clinical relevance, the association between motor symptom severity and urinary dysfunction remains poorly understood. This study aimed to elucidate this relationship using validated clinical questionnaires to assess urinary symptoms.

**Methods:**

We conducted a single-center, retrospective cross-sectional study including 223 patients with PD who visited a university hospital between September 2023 and February 2024. Urinary dysfunction was evaluated using the Overactive Bladder Symptom Score (OABSS) and International Prostate Symptom Score (IPSS), comprising the symptom (IPSS-symptom score; Q1–7) and satisfaction (IPSS-satisfaction score; Q8) scores. We analyzed the changes in urinary symptoms, overall satisfaction, and prodromal symptoms across the Hoehn and Yahr (HY) stages. Patients were divided into early (HY ≤ 2) and late (HY > 2) groups to assess early urinary symptom changes.

**Results:**

The OABSS, IPSS-symptom score, and IPSS-satisfaction score significantly increased with advancing HY stage. Compared to the early group, the late group exhibited significantly higher OABSS (*p* = 0.015), IPSS-symptom scores (*p* = 0.002), and IPSS-satisfaction scores (*p* < 0.001). Subgroup analysis of the IPSS revealed that storage symptoms compared with voiding symptoms correlated more strongly with motor severity.

**Conclusion:**

Our study provides evidence that urinary dysfunction intensifies with motor symptom progression in PD. These findings highlight the importance of early detection and proactive management of urinary symptoms in patients with PD to enhance their overall quality of life.

## Introduction

Urinary dysfunction is one of the most prevalent non-motor symptoms in patients with Parkinson’s disease (PD). It can manifest in the early stages of the disease and tends to worsen with disease progression, posing a substantial burden on patients’ daily functioning and quality of life. Among the various urinary symptoms, overactive bladder (OAB), characterized by urgency with or without urgency incontinence and typically accompanied by frequency and nocturia, affects up to 70% of patients with PD (Araki et al., 2000; Campos-Sousa et al., 2003; Sakakibara et al., 2010). The pathophysiological mechanism underlying OAB in PD is detrusor overactivity, resulting from a disrupted brain–bladder regulatory pathway. The degeneration of the frontal-basal ganglia D1 dopaminergic circuit, which normally provides inhibitory control over the micturition reflex, plays a key role in this dysfunction (Sakakibara et al., 2014). Recent neuroimaging and pathological studies have further supported that nigrostriatal dopaminergic loss contributes to impaired bladder inhibition through disrupted connectivity between the basal ganglia, pontine micturition center, and prefrontal cortex (Chen et al., 2020; Qin et al., 2021).

With the increasing older adult population, understanding the relationship between urinary dysfunction and disease severity in PD has become increasingly important, not only for optimizing clinical management but also for improving patients’ overall quality of life. Previous studies have reported that urinary symptoms tend to become more prevalent and severe as PD progresses (Winge and Nielsen, 2012; Mito et al., 2016). These symptoms may even precede motor manifestations by occurring in the prodromal phase and are associated with greater motor disability, more extensive dopaminergic denervation, and reduced quality of life (Uchiyama et al., 2011; Durcan et al., 2019; Martinez-Ramirez et al., 2020). Although prior studies have suggested a link between disease severity and urinary symptoms, the results remain inconsistent and underpowered, particularly in terms of sex-specific patterns and early-stage characterization (Campos-Sousa et al., 2003; Defreitas et al., 2003; Hobson et al., 2003). Relatively few studies have focused on the relationship between motor severity and urinary dysfunction in PD. Therefore, we aimed to investigate this association using validated clinical questionnaires and characterize the presence and progression of early urinary symptoms in patients with PD.

## Materials and methods

### Participants and clinical assessment

In this single-center retrospective study, we enrolled 223 patients with PD who visited the neurology clinic at Dongsan Hospital, Daegu, Republic of Korea, between September 2023 and February 2024. The clinical diagnosis of PD was established based on the Movement Disorder Society Clinical Diagnostic Criteria for PD (Postuma et al., 2015). Motor symptom severity was assessed using the Hoehn and Yahr (HY) stages during the “on” state (Hoehn and Yahr, 1967).

Demographic and clinical data, including age at disease onset, sex, disease duration, and the presence of PD prodromal symptoms, such as hyposmia, rapid eye movement sleep behavior disorder (RBD), and constipation, were obtained from medical records. RBD was identified by the presence of dream-enactment behaviors reported by the patient or bed partner, and constipation was defined as bowel movements fewer than three times per week or the regular use of laxatives, based on information obtained from clinical interviews or medical record documentation. Comorbidities, including hypertension, diabetes, and benign prostatic hyperplasia, and medications patients were taking at the time of the evaluation, including levodopa, dopamine agonists, anticholinergics, and amantadine, were also thoroughly investigated. Additionally, the levodopa equivalent daily dose (LEDD) was calculated for each patient (Schade et al., 2020). Patients with Parkinson-plus syndromes [e.g., multiple system atrophy (MSA), progressive supranuclear palsy, and corticobasal syndrome] or secondary parkinsonism (e.g., vascular or drug-induced parkinsonism) were excluded. Patients who had undergone urological surgery, had a history of renal or urinary tract infections within the past 6 months, or had been diagnosed with malignancies were also excluded.

This study was approved by the Institutional Review Board of Dongsan Hospital, Keimyung University, Daegu, Republic of Korea (IRB No. 2025-01-007). Written informed consent was obtained from all the participants.

### Questionnaire evaluation

All patients were assessed for urinary dysfunction using two validated questionnaires, the Korean version of the OAB Symptom Score (OABSS) and the International Prostate Symptom Score (IPSS) questionnaires, regardless of whether they had subjective urinary complaints (Choi et al., 1996; Jeong et al., 2011). These questionnaires are easily administered in an outpatient clinic setting and have been widely used in previous studies to evaluate urinary symptoms in patients with PD (Araki et al., 2000; Mito et al., 2016; Jia et al., 2020). Both the OABSS and IPSS questionnaires were self-administered by the patients under supervision.

The OABSS consists of four questions regarding daytime frequency (Question 1, Q1), nocturia (Q2), urgency (Q3), and urgency incontinence (Q4) and evaluates relevant symptoms from the patient’s perspective. The total OABSS score was calculated by summing the scores for all four items. An OAB diagnosis is defined as a total score of ≥3, with an urgency score (Q3) ≥ 2 and its severity is classified as mild (total score ≤ 5), moderate (6–11), or severe (≥12) (Homma et al., 2006; Yamaguchi et al., 2009). The IPSS is based on answers to seven questions concerning urinary symptoms (IPSS-symptoms score) including incomplete emptying (Q1), frequency (Q2), intermittency (Q3), urgency (Q4), weak stream (Q5), straining (Q6), and nocturia (Q7) and one question concerning general satisfaction with urinary conditions (IPSS-satisfaction score; Q8). The IPSS-symptom scores (Q1–7) were classified as mild (0–7), moderate (8–19), or severe (20–35). Response to general satisfaction (Q8) range from “delighted” (0) to “terrible” (6) (Barry et al., 1992).

We investigated the trends in urinary symptoms, general satisfaction, and prodromal symptoms across the HY stages. For a more detailed analysis, the IPSS was subdivided into storage (Q2, Q4, and Q7) and voiding (Q1, Q3, Q5, and Q6) symptoms. To evaluate the progression of urinary symptoms according to disease severity, changes in the questionnaire scores were compared at each HY stage, using HY 1 as the baseline.

For further subgroup analysis, patients were classified into the early (HY ≤ 2) and late (HY > 2) groups, and an additional comparison was made between the very early (HY 1) and more advanced (HY ≥ 2) groups to detect symptom changes from the earliest phase of PD. The two subgroup comparisons were designed to represent different clinical perspectives on disease progression. The early–late grouping reflects the conventional distinction between early and later stages of PD, whereas the very early–more advanced comparison aimed to determine whether urinary dysfunction may already emerge at the earliest stage of PD. Although overlapping, these definitions are complementary in addressing both conventional staging and the earliest phase of urinary symptom development. To assess potential sex-related differences in urinary symptoms, subgroup analyses were conducted separately for male and female patients.

### Statistical analysis

Continuous variables are described as means and standard deviations, and categorical variables are described as frequencies and percentages. To compare variables across the HY stages, one-way analysis of variance was used for continuous variables, and the chi-square or Fisher’s exact test was applied for categorical variables. When the overall test results reached statistical significance, Dunnett’s *post-hoc* tests were performed for each stage (HY 2–5) compared to the baseline stage (HY 1), and adjusted *p*-values were presented accordingly. To assess changes in questionnaire scores across the HY stages, the mean OABSS and IPSS scores were plotted by HY stage using line graphs. If statistically significant differences were observed, the corresponding *p*-values were annotated. For group comparisons, independent two-sample *t*-test were used to compare questionnaire scores between the early (HY ≤ 2) and late (HY > 2) groups, as well as between the very early (HY 1) and more advanced (HY ≥ 2) groups. All statistical tests were two-tailed, and the α level was set at *p* < 0.05. Analyses were performed using SAS version 9.4 (SAS Institute Inc., Cary, NC, USA) and R version 4.3.2 (R Foundation for Statistical Computing, Vienna, Austria).

## Results

### Demographic and clinical characteristics across HY stages

Clinical characteristics, such as age at disease onset, disease duration, and LEDD, increased significantly with advancing HY stage (*p* < 0.001 for all). Although the sex distribution showed a statistically significant difference across stages (*p* = 0.005), no consistent trend was observed. The prevalence of comorbidities, such as hypertension, diabetes mellitus, and benign prostatic hyperplasia, did not differ significantly among the groups. Among the prodromal symptoms, only constipation was significantly associated with the HY stage (*p* = 0.004), whereas hyposmia and RBD showed no significant association (Table 1).

**TABLE 1:** Demographic and clinical characteristics by Hoehn and Yahr stages.

	Stage 1 (*n* = 53)	Stage 2 (*n* = 90)	Stage 3 (*n* = 36)	Stage 4 (*n* = 36)	Stage 5 (*n* = 8)	*P*-value
Age at onset (years)	65.4 ± 8.8	68.0 ± 8.6	70.7 ± 9.7^†^	75.4 ± 6.5^†^	71.8 ± 6.5	**<0.001^a^**
Sex (M/F)	30/23	40/50	19/17	8/28^†^	1/7	**0.005^b^**
Disease duration (years)	2.8 ± 1.8	4.1 ± 2.7^†^	6.9 ± 4.1^†^	10.1 ± 5.0^†^	10.5 ± 3.6^†^	**<0.001^a^**
LEDD (mg/day)	242.6 ± 168.1	491.1 ± 332.8^†^	722.7 ± 420.2^†^	927.4 ± 387.8^†^	1156.6 ± 360.4^†^	**<0.001^a^**
Hyposmia, *n* (%)	28 (52.8)	52 (57.8)	25 (69.4)	16 (44.4)	2 (25.0)	0.093^b^
RBD, *n* (%)	26 (49.1)	47 (52.2)	20 (55.6)	22 (61.1)	3 (37.5)	0.704^b^
Constipation, *n* (%)	25 (47.2)	62 (68.9)^†^	22 (61.1)	30 (83.3)^†^	7 (87.5)	**0.004^b^**
HTN, *n* (%)	14 (26.4)	34 (37.8)	15 (41.7)	14 (38.9)	4 (50.0)	0.474^b^
DM, *n* (%)	7 (13.2)	18 (20.0)	9 (25.0)	7 (19.4)	2 (25.0)	0.697^b^
BPH, *n* (%)	8 (15.1)	11 (12.2)	1 (2.8)	6 (16.7)	0 (0)	0.651^c^

HY, Hoehn and Yahr; LEDD, Levodopa Equivalent Daily Dose; RBD, Rapid eye movement sleep Behavior Disorder; HTN, Hypertension; DM, Diabetes Mellitus; BPH, Benign Prostatic Hyperplasia. Data are presented as mean ± standard deviation. Bold values denote overall statistical significance (*p* < 0.05). *P*-values were calculated using one-way ANOVA*^a^*, the chi-square test*^b^*, or Fisher’s exact test*^c^*, depending on the variable type. ^†^Adjusted *p* < 0.05 for Dunnett’s *post hoc* comparisons versus HY Stage 1.

### Changes in OABSS and IPSS scores across HY Stages

**FIGURE 1:**
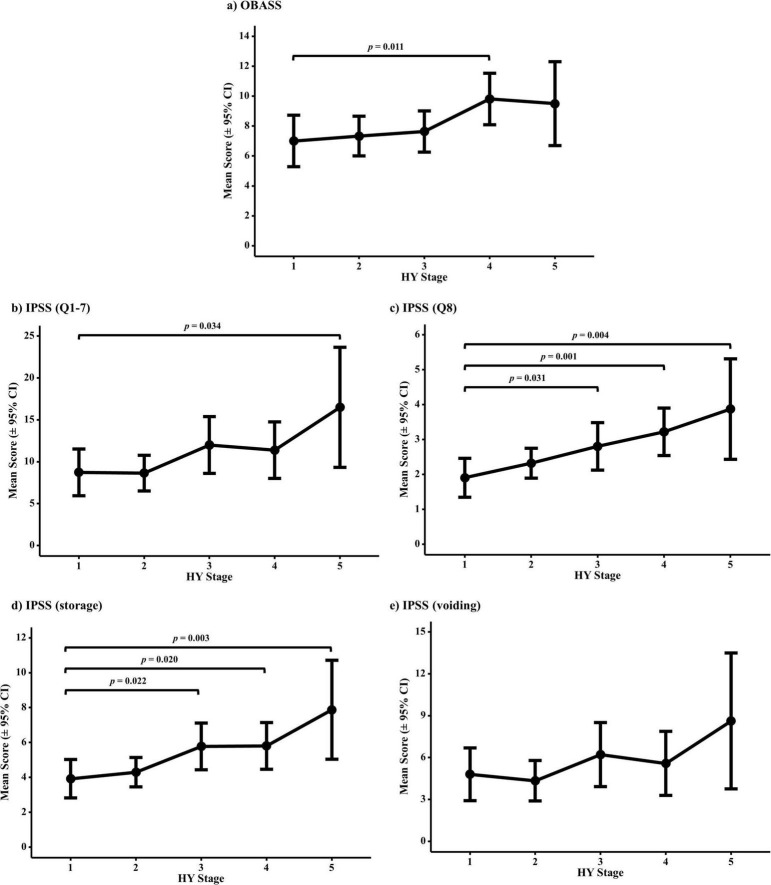
Comparison of mean scores of each questionnaire item across HY stages. Scores were analyzed using one-way ANOVA with Dunnett’s *post-hoc* test, using HY stage 1 as the reference. (a) OABSS, (b) IPSS (Q1–7), (c) IPSS (Q8), (d) IPSS storage subscores, and (e) IPSS voiding subscores. All *p*-values represent adjusted values from Dunnett’s test. Significant differences compared to HY stage 1 are indicated, with an adjusted *p* < 0.05. Error bars represent the 95% confidence intervals.

As the HY stage increased, significant differences were observed in the total OABSS, IPSS-symptom score (Q1–7), IPSS-satisfaction score (Q8), and IPSS-storage sub-scores across stages. In the *post hoc* analysis, the OABSS was significantly higher at stage 4 than at stage 1 (adjusted *p* = 0.011). Only stage 5 demonstrated a statistically significant increase in the subtotal IPSS (Q1–7), compared with that observed in stage 1 (adjusted *p* = 0.034). IPSS-satisfaction scores (Q8), where higher values indicate greater dissatisfaction, significantly increased at stages 3 (adjusted *p* = 0.031), 4 (adjusted *p* = 0.001), and 5 (adjusted *p* = 0.004) compared with stage 1. Subgroup analysis of the IPSS revealed a significant upward trend in the association of storage symptoms with advancing HY stage, with mean scores significantly higher at stages 3 (adjusted *p* = 0.022), 4 (adjusted *p* = 0.019), and 5 (adjusted *p* = 0.003) than at stage 1. In contrast, voiding symptom scores did not differ significantly across the HY stages, even with progression to stage 5 (Figure 1, Table 2, and Supplementary Table 1). This finding remained unchanged after adjustment for the use of anticholinergics and amantadine (Supplementary Table 2).

**TABLE 2:** Overactive Bladder Symptom Score (OABSS) and IPSS subscores by HY stages.

	Stage 1 (*n* = 53)	Stage 2 (*n* = 90)	Stage 3 (*n* = 36)	Stage 4 (*n* = 36)	Stage 5 (*n* = 8)	*P*-value
OABSS	7.0 ± 3.3	7.3 ± 2.4	7.6 ± 2.4	9.8 ± 2.8^†^	9.5 ± 2.0	**<0.001**
IPSS (Q1–7)	8.7 ± 8.4	8.6 ± 6.7	12.0 ± 7.8	11.4 ± 9.0	16.5 ± 9.7^†^	**0.012**
IPSS (Q8)	1.9 ± 1.5	2.3 ± 1.4	2.8 ± 1.8^†^	3.2 ± 1.6^†^	3.9 ± 2.2^†^	**<0.001**
IPSS (storage)	3.9 ± 3.1	4.3 ± 2.6	5.8 ± 3.1^†^	5.8 ± 3.9^†^	7.9 ± 3.3^†^	**<0.001**
IPSS (voiding)	4.8 ± 5.7	4.3 ± 4.7	6.2 ± 5.4	5.6 ± 5.6	8.6 ± 6.6	0.118

OABSS, Overactive Bladder Symptom Score; IPSS, International Prostate Symptom Score; HY, Hoehn and Yahr. The IPSS (Q1–7) reflects urinary symptoms, and Q8 represents overall satisfaction. The OABSS and IPSS (Q1–7) were calculated as a sum. IPSS (storage) is the average of Q2, 4, and 7, and IPSS (voiding) is the average of Q1, 3, 5, and 6. Data are presented as mean ± standard deviation. Bold values denote overall statistical significance (*p* < 0.05). ^†^Adjusted *p* < 0.05 for Dunnett’s *post hoc* comparisons versus HY Stage 1.

### Comparison of OABSS and IPSS by HY-defined groups

In a comparative analysis between the early and late groups, the OABSS (*p* = 0.015), IPSS-symptom score (Q1–7) (*p* = 0.002), and IPSS-satisfaction score (Q8) (*p* < 0.001) were significantly higher in the late group. When comparing the very early and more advanced groups, only the IPSS-satisfaction score (Q8) showed a significant difference (*p* = 0.002). Subgroup analysis of the IPSS indicated that the storage symptom scores were significantly higher in both the late and more advanced groups than in their respective counterpart groups (*p* < 0.001 and *p* = 0.020, respectively). Voiding symptom scores were significantly higher in the late group than in the early group; however, no significant difference was observed between the very early and more advanced groups (Table 3). The demographic and clinical characteristics of the groups based on the HY stage are presented in Supplementary Table 3.

**TABLE 3:** Groupwise comparison of OABSS and IPSS subscores by HY stage.

	Early (*n* = 143)	Late (*n* = 80)	*P*-value	Very early (*n* = 53)	More advanced (*n* = 170)	*P*-value
OABSS	7.2 ± 2.7	8.6 ± 2.7	**0.015**	7.0 ± 3.2	8.1 ± 2.6	0.132
IPSS (Q1–7)	8.7 ± 7.4	12.2 ± 8.6	**0.002**	8.7 ± 8.4	10.3 ± 7.8	0.211
IPSS (Q8)	2.2 ± 1.5	3.1 ± 1.7	**<0.001**	1.9 ± 1.5	2.7 ± 1.6	**0.002**
IPSS (storage)	4.2 ± 2.8	6.0 ± 3.5	**<0.001**	3.9 ± 3.1	5.1 ± 3.2	**0.020**
IPSS (voiding)	4.5 ± 5.1	6.2 ± 5.6	**0.023**	4.8 ± 5.7	5.2 ± 5.2	0.639

OABSS, Overactive Bladder Symptom Score; IPSS, International Prostate Symptom Score; HY, Hoehn and Yahr. Early, HY 1–2; Late, HY 3–5; Very Early, HY 1; More Advanced, HY 2–5. The IPSS (Q1–7) relates to urinary symptoms, and the IPSS (Q8) relates to overall satisfaction. The IPSS (storage) is the average of Q2, 4, and 7, and the IPSS (voiding) is the average of Q1, 3, 5, and 6. Data are presented as mean ± standard deviation. *P*-values were obtained using independent two-sample *t*-test. Bold values denote statistical significance (*p* < 0.05).

### Sex-stratified analysis of OABSSs and IPSSs

The chi-square test revealed a significant association between HY stage and sex (*p* = 0.005). *Post hoc* analysis further showed a significant difference specifically in HY stage 4 between male and female patients (*p* = 0.0018). In the analyses stratified by sex, both male and female patients showed significantly higher IPSS subscores (Q1–7 and Q8) in the late group than in the early group. However, male patients exhibited a significant increase in only the storage symptom score, whereas female patients demonstrated a significant increase in both storage and voiding symptom scores. The detailed sex results are presented in Supplementary Table 4.

## Discussion

Urinary dysfunction, including OAB, is a prevalent non-motor symptom in patients with PD, substantially affecting their quality of life. This study aimed to assess the relationship between disease severity and urinary dysfunction in patients with PD using two validated clinical assessment tools. Our findings demonstrated a clear correlation between increased motor symptom severity and worsened urinary dysfunction, as evidenced by a significant increase in both OABSSs and IPSSs with advancing HY stage. These findings are consistent with those of previous studies, indicating that urinary dysfunction becomes increasingly prevalent and severe as PD progresses (Winge et al., 2006; Winge and Nielsen, 2012; Mito et al., 2016; Buchman et al., 2017). A recent study characterized urinary symptoms across PD subtypes and found no significant differences in lower urinary tract symptoms among them (Gubbiotti et al., 2025). In contrast, our stage-based analysis demonstrated a progressive increase in urinary symptom severity with advancing HY stage, suggesting that disease progression, rather than motor phenotype, is a key determinant of urinary dysfunction in PD.

Urinary symptoms in PD, particularly OAB, are believed to arise from neurodegenerative changes in the central micturition pathway. In particular, the basal ganglia and frontal lobes, which are critical for voluntary bladder control, are implicated. Although the exact pathophysiology remains unclear, evidence has suggested that degeneration of the nigrostriatal dopaminergic system impairs the inhibitory control of the micturition reflex, resulting in detrusor overactivity (Sakakibara et al., 2014; Qin et al., 2021). Functional neuroimaging studies have demonstrated reduced dopamine transporter activity in patients with PD who have lower urinary tract symptoms, further supporting this mechanism (Qin et al., 2021). Our research reinforces this pathophysiological link by demonstrating that urinary dysfunction is directly associated with the progression of motor symptoms, potentially reflecting cumulative dopaminergic degeneration in brain regions involved in bladder control.

A key finding of our study was that the OAB levels increased significantly with PD progression. This finding is consistent with previous reports that these symptoms are common in patients with advanced PD (Winge and Nielsen, 2012; Yeo et al., 2012; Vaughan et al., 2013; Li et al., 2022). Nocturia and incontinence are particularly burdensome and clinically significant, as they tend to have the most direct impact on patients’ daily functioning. Nocturia can severely disrupt sleep architecture, contribute to increased fatigue, and adversely affect quality of life (Vaughan et al., 2013; Diaconu et al., 2023). As motor and autonomic dysfunction worsens with PD progression, patients may encounter more frequent and less controllable nocturnal voiding episodes. These symptoms do not only contribute to physical discomfort but may also exacerbate neuropsychiatric symptoms, thereby increasing the overall disease burden.

Our study revealed significant differences in both the OABSS and IPSS between the early and late groups, underlining the importance of identifying urinary symptoms early in the disease course. This finding aligns with previous research suggesting that while OAB symptoms can be present even in the early stages of PD, their severity tends to increase as motor symptoms progress (Liu et al., 2015; Kim et al., 2022). Furthermore, patients in more advanced stages often present with more complex symptom profiles, which may obscure the initial manifestations of urinary dysfunction. Although originally developed for non-neurological populations, the OABSS has been proven as a useful tool for evaluating urinary symptoms in patients with PD through the identification of early symptom burden and monitoring of treatment-related changes (Jeong et al., 2011; Liu et al., 2015; Mito et al., 2016; Jia et al., 2020). In addition, our data showed that patient satisfaction and storage symptoms were distinguishable at the very early stage, suggesting that urinary discomfort and storage-related dysfunction may begin in the initial phase of PD. These findings reinforce the need to consider urinary symptoms as meaningful clinical features, even in the early stages of PD (Uchiyama et al., 2011; Martinez-Ramirez et al., 2020).

We also found that storage symptoms, as measured by the IPSS, compared with voiding symptoms were more strongly associated with motor severity. In contrast, voiding symptoms did not significantly correlate with the HY stage, even in the advanced stages. Previous studies have reported that in PD, storage symptoms are generally more prevalent than voiding symptoms and that detrusor underactivity has been associated with motor impairment in patients with PD (Uchiyama et al., 2011; Yeo et al., 2012; Sakakibara et al., 2014; Liu et al., 2015; Mito et al., 2016). Most of these studies assessed PD populations without stratification by sex, and the relationship between urinary symptoms and disease severity was largely considered irrespective of sex.

In contrast, our sex-stratified analysis revealed that while storage symptoms were consistently associated with motor severity in both sexes, voiding symptoms significantly increased in only female patients in the advanced stages. These differences are likely attributable not merely to structural or hormonal factors but to neurogenic mechanisms related to PD progression. Neurodegeneration within the pontine micturition center and basal ganglia circuits can impair detrusor contractility and coordination between the detrusor and urethral sphincter, resulting in detrusor underactivity or detrusor–sphincter dyssynergia. In women, the relatively low baseline urethral resistance may initially mask such dysfunction; however, as neurodegeneration advances, the diminished detrusor contractile force may manifest as clinically significant voiding difficulty. The interaction between PD-related neurodegeneration and sex-specific physiological characteristics may therefore underlie the worsening of voiding symptoms observed in female patients with advanced PD. Furthermore, voiding symptoms, which are prominent in the early stages of the disease, are more characteristic of MSA, possibly helping to differentiate it from PD (Yeo et al., 2012; Sakakibara et al., 2014; Jiang et al., 2025). Therefore, the presence of predominant voiding dysfunction in patients in the early stage may be a clinical clue for differentiating PD from other neurodegenerative disorders, such as MSA, warranting further evaluation, such as autonomic function testing.

Another interesting finding is the association between constipation and urinary dysfunction. Constipation is a well-recognized prodromal symptom of PD and is linked to both motor and non-motor manifestations (Durcan et al., 2019). Previous studies have suggested that constipation and urinary symptoms may share overlapping pathophysiological mechanisms involving autonomic nervous dysfunction that affects both gastrointestinal and lower urinary tract regulation (Chen et al., 2020; Pfeiffer, 2020). Our findings support these previous suggestions, and although our analysis was limited to constipation, the results suggest that certain non-motor symptoms with similar neurophysiological bases may warrant integrated clinical consideration.

Our study has several limitations. First, its retrospective cross-sectional design limited causal inferences regarding the relationship between motor severity and urinary dysfunction. Longitudinal studies are needed to clarify the progression of urinary symptoms across disease stages. Second, because this was a single-center study, the findings may not be generalizable to other populations with PD. Larger multicenter studies are necessary to validate and extend our findings. Third, although we utilized validated patient-reported questionnaires, these are inherently subject to recall bias and may have led to the underreporting of symptoms. Incorporating objective assessments, such as urodynamic studies, could provide more precise insights into the mechanisms underlying urinary dysfunction in PD. Fourth, despite our efforts to exclude various confounders, we could not fully account for the potential effects of anticholinergic medications and amantadine. These medications are commonly prescribed, but possess antimuscarinic properties that may exacerbate urinary symptoms. Future studies should consider controlling for or excluding the use of these medications to isolate patients with disease-related urinary dysfunction better.

## Conclusion

Our study provides further evidence of the increasing severity of urinary dysfunction, especially storage-related symptoms such as OAB, with the progression of PD. Early recognition and management of urinary dysfunction in PD are essential to enhance patients’ quality of life. Furthermore, early identification of urinary dysfunction may help distinguish PD from atypical parkinsonism, such as MSA, in which voiding symptoms typically appear at an earlier stage. Future research should aim to clarify the mechanisms underlying urinary dysfunction in PD and explore targeted therapeutic strategies to address this clinically important aspect of the disease.

## Data Availability

The raw data supporting the conclusions of this article will be made available by the authors, without undue reservation.
