# Supplementary material for: Association between motor symptom severity and urinary dysfunction in Parkinson’s disease: a retrospective study
Source: Front Aging Neurosci. 2025 Dec 12;17:1688656. doi: 10.3389/fnagi.2025.1688656 (PMC12740936; doi:10.3389/fnagi.2025.1688656)
Supplement: Supplementary file 1 [file Data_Sheet_1.docx]

**Supplementary Table 1.** OABSS and IPSS subscores by HY stages

* Sensitivity analysis excluding HY stage 5

|  | Stage 1 (n = 53) | Stage 2 (n = 90) | Stage 3 (n = 36) | Stage 4 (n = 36) | *p-value* |
| --- | --- | --- | --- | --- | --- |
| OABSS | 7.0 ± 3.3 | 7.3 ± 2.4 | 7.6 ± 2.4 | 9.8 ± 2.8^†^ | **0.012** |
| IPSS (Q1–7) | 8.7 ± 8.4 | 8.6 ± 6.7 | 12.0 ± 7.8 | 11.4 ± 9.0 | 0.063 |
| IPSS (Q8) | 1.9 ± 1.5 | 2.3 ± 1.4 | 2.8 ± 1.8^†^ | 3.2 ± 1.6^†^ | **0.001** |
| IPSS (Storage) | 3.9 ± 3.1 | 4.3 ± 2.6 | 5.8 ± 3.1^†^ | 5.8 ± 3.9^†^ | **0.004** |
| IPSS (Voiding) | 4.8 ± 5.7 | 4.3 ± 4.7 | 6.2 ± 5.4 | 5.6 ± 5.6 | 0.279 |

OABSS, Overactive Bladder Symptom Score; IPSS, International Prostate Symptom Score; HY, Hoehn and Yahr.
The IPSS (Q1–7) reflects urinary symptoms, and Q8 represents overall satisfaction.
The OABSS and IPSS (Q1–7) were calculated as a sum.
IPSS (storage) is the average of Q2, 4, and 7, and IPSS (voiding) is the average of Q1, 3, 5, and 6.
Data are presented as mean ± standard deviation.
Bold values denote overall statistical significance (*p* < 0.05).
^†^ Adjusted *p* < 0.05 for Dunnett’s post-hoc comparisons versus HY Stage 1.

**Supplementary Table 2.** OABSS and IPSS subscores by HY stages

* Multivariate analysis adjusted for anticholinergics and amantadine

|  | Stage 1 (n = 53) | Stage 2 (n = 90) | Stage 3 (n = 36) | Stage 4 (n = 36) | Stage 5 (n = 8) | *p-value* |
| --- | --- | --- | --- | --- | --- | --- |
| OABSS | 7.0 ± 3.3 | 7.3 ± 2.4 | 7.6 ± 2.4 | 9.8 ± 2.8^†^ | 9.5 ± 2.0 | **0.012** |
| IPSS (Q1–7) | 8.7 ± 8.4 | 8.6 ± 6.7 | 12.0 ± 7.8 | 11.4 ± 9.0 | 16.5 ± 9.7^†^ | **0.028** |
| IPSS (Q8) | 1.9 ± 1.5 | 2.3 ± 1.4 | 2.8 ± 1.8^†^ | 3.2 ± 1.6^†^ | 3.9 ± 2.2^†^ | **0.001** |
| IPSS (Storage) | 3.9 ± 3.1 | 4.3 ± 2.6 | 5.8 ± 3.1^†^ | 5.8 ± 3.9^†^ | 7.9 ± 3.3^†^ | **0.001** |
| IPSS (Voiding) | 4.8 ± 5.7 | 4.3 ± 4.7 | 6.2 ± 5.4 | 5.6 ± 5.6 | 8.6 ± 6.6 | 0.205 |

OABSS, Overactive Bladder Symptom Score; IPSS, International Prostate Symptom Score; HY, Hoehn and Yahr.
The IPSS (Q1–7) reflects urinary symptoms, and Q8 represents overall satisfaction.
The OABSS and IPSS (Q1–7) were calculated as a sum.
IPSS (storage) is the average of Q2, 4, and 7, and IPSS (voiding) is the average of Q1, 3, 5, and 6.
Data are presented as mean ± standard deviation.
Bold values denote overall statistical significance (*p* < 0.05).
^†^ Adjusted *p* < 0.05 for Dunnett’s post-hoc comparisons versus HY Stage 1.

**Supplementary Table 3.** Groupwise comparison of demographic and clinical characteristics by HY stages

|  | Early  (n = 143) | Late  (n = 80) | *p*-value | Very early  (n = 53) | More advanced  (n = 170) | *p*-value |
| --- | --- | --- | --- | --- | --- | --- |
| Age at onset (years) | 67.0 ± 8.7 | 72.9 ± 8.3 | **<0.001**^a^ | 65.4 ± 8.8 | 70.3 ± 8.8 | **<0.005**^a^ |
| Sex (M/F) | 70/73 | 28/52 | **0.044**^b^ | 30/23 | 68/102 | **0.034**^b^ |
| Disease duration (years) | 3.6 ± 2.5 | 8.7 ± 4.7 | **<0.001**^a^ | 2.8 ± 1.8 | 6.3 ± 4.4 | **<0.001**^a^ |
| LEDD (mg/day) | 399.0 ± 307.1 | 858.4 ± 419.6 | **<0.001**^a^ | 242.5 ± 168.1 | 663.9 ± 417.7 | **<0.001**^a^ |
| Hyposmia, n (%) | 80 (55.9) | 43 (53.8) | 0.752^b^ | 28 (52.8) | 95 (56.0) | 0.697^b^ |
| RBD, n (%) | 73 (51.1) | 45 (56.3) | 0.455^b^ | 26 (49.1) | 92 (54.1) | 0.519^b^ |
| Constipation, n (%) | 87 (60.8) | 59 (73.8) | 0.052^b^ | 25 (47.2) | 121 (71.2) | **0.001**^b^ |
| HTN, n (%) | 48 (33.6) | 33 (41.3) | 0.252^b^ | 14 (26.4) | 67 (39.4) | 0.086^b^ |
| DM, n (%) | 25 (17.5) | 18 (22.5) | 0.362^b^ | 7 (13.2) | 36 (21.2) | 0.200^b^ |
| BPH, n (%) | 14 (9.8) | 4 (5.0) | 0.208^b^ | 4 (7.6) | 14 (8.2) | 0.999^c^ |

HY, Hoehn and Yahr; LEDD, Levodopa Equivalent Daily Dose; RBD, Rapid eye movement sleep Behavior Disorder; HTN, Hypertension; DM, Diabetes Mellitus; BPH, Benign Prostatic Hyperplasia.
Early: HY 1–2; Late: HY 3–5; Very Early: HY 1; More Advanced: HY 2–5.
Data are presented as mean ± standard deviation.
P-values were calculated using an independent two-sample *t*-test^a^, chi-square test^b^, or Fisher’s exact test^c^, depending on the variable type.
Bold values denote overall statistical significance (*p* < 0.05).

**Supplementary Table 4.** Sex-stratified comparison of OABSS and IPSS subscores by HY stages

* Male (n = 98)

|  | Early (n = 70) | Late (n = 28) | *p*-value | Very early (n = 30) | More advanced (n = 68) | *p*-value |
| --- | --- | --- | --- | --- | --- | --- |
| OABSS | 6.6 ± 1.9 | 7.8 ± 2.3 | 0.076 | 6.4 ± 1.7 | 7.4 ± 2.2 | 0.261 |
| IPSS (Q1–7) | 10.2 ± 7.5 | 14.6 ± 7.9 | **0.011** | 9.5 ± 7.8 | 12.3 ± 7.8 | 0.099 |
| IPSS  (Q8) | 2.4 ± 1.5 | 3.2 ± 1.7 | **0.020** | 2.0 ± 1.5 | 2.8 ± 1.6 | **0.020** |
| IPSS (Storage) | 4.2 ± 2.6 | 6.4 ± 3.4 | **<0.001** | 3.9 ± 2.7 | 5.3 ± 3.1 | **0.040** |
| IPSS (Voiding) | 6.0 ± 5.4 | 8.3 ± 5.2 | 0.061 | 5.6 ± 5.7 | 5.2 ± 5.2 | 0.639 |

* Female (n = 125)

|  | Early (n = 73) | Late (n = 52) | *p*-value | Very early (n = 23) | More advanced (n = 102) | *p*-value |
| --- | --- | --- | --- | --- | --- | --- |
| OABSS | 7.8 ± 3.3 | 9.3 ± 2.8 | 0.101 | 8.0 ± 5.0 | 8.7 ± 2.8 | 0.750 |
| IPSS (Q1–7) | 7.2 ± 6.9 | 10.9 ± 8.7 | **0.011** | 7.7 ± 9.2 | 9.0 ± 7.6 | 0.508 |
| IPSS  (Q8) | 2.0 ± 1.5 | 3.1 ± 1.8 | **<0.001** | 1.8 ± 1.5 | 2.6 ± 1.7 | **0.027** |
| IPSS (Storage) | 4.1 ± 3.0 | 5.8 ± 3.6 | **0.006** | 4.0 ± 3.7 | 5.0 ± 3.3 | 0.180 |
| IPSS (Voiding) | 3.1 ± 4.4 | 5.1 ± 5.5 | **0.031** | 3.8 ± 5.6 | 4.0 ± 4.9 | 0.884 |

OABSS, Overactive Bladder Symptom Score; IPSS, International Prostate Symptom Score; HY, Hoehn and Yahr
Early: HY 1–2; Late: HY 3–5; Very Early: HY 1; More Advanced: HY 2–5.
The IPSS (Q1–7) relates to urinary symptoms, and the IPSS (Q8) relates to overall satisfaction.
The IPSS (storage) is the average of Q2, 4, and 7, and the IPSS (voiding) is the average of Q1, 3, 5, and 6.
Data are presented as mean ± standard deviation.
*P*-values were obtained using independent two-sample *t*-test.
Bold values denote statistical significance (*p* < 0.05).
